# The Role of Nutrition and Other Lifestyle Patterns in Mortality Risk in Older Adults with Multimorbidity

**DOI:** 10.3390/nu17050796

**Published:** 2025-02-25

**Authors:** Chao Dong, Karen A. Mather, Henry Brodaty, Perminder S. Sachdev, Julian Trollor, Fleur Harrison, Dana Bliuc, Rebecca Ivers, Joel Rhee, Zhaoli Dai

**Affiliations:** 1 Centre for Healthy Brain Ageing (CHeBA), Discipline of Psychiatry and Mental Health, School of Clinical Medicine, Faculty of Medicine and Health, University of New South Wales, Sydney 2052, Australia; chao.dong1@unswalumni.com (C.D.); karen.mather@unsw.edu.au (K.A.M.); h.brodaty@unsw.edu.au (H.B.); p.sachdev@unsw.edu.au (P.S.S.); f.harrison@unsw.edu.au (F.H.); 2 Australia National Centre of Excellence in Intellectual Disability Health, School of Psychiatry, Faculty of Medicine and Health, University of New South Wales, Sydney 2052, Australia; j.trollor@unsw.edu.au; 3 School of Population Health, Faculty of Medicine and Health, University of New South Wales, Sydney 2052, Australia; d.bliuc@unsw.edu.au (D.B.); rebecca.ivers@unsw.edu.au (R.I.); 4 Discipline of General Practice, School of Clinical Medicine, Faculty of Medicine and Health, University of New South Wales, Sydney 2052, Australia; j.rhee@unsw.edu.au; 5 School of Pharmacy, Faculty of Medicine and Health, University of Sydney, Sydney 2006, Australia

**Keywords:** diet and nutrition, physical activity, sleep, social engagement, comorbid conditions

## Abstract

**Background:** Limited research has examined how older adults’ lifestyles intersect with multimorbidity to influence mortality risk. **Methods:** In this community-dwelling prospective cohort, the Sydney Memory and Ageing Study, principal component analysis was used to identify lifestyle patterns using baseline self-reported data on nutrition, lifestyle factors, and social engagement activities. Multimorbidity was defined by self-reported physician diagnoses. Multivariable logistic regression was used to estimate odds ratios (ORs) for multimorbidity cross-sectionally, and Cox proportional hazards models were used to assess hazard ratios (HRs) for mortality risk longitudinally. **Results:** Of 895 participants (mean age: 78.2 years; 56.3% female) with complete lifestyle data, 597 had multimorbidity. Two distinct lifestyle patterns emerged: (i) a nutrition pattern characterised by higher intakes of protein, fibre, iron, zinc, magnesium, potassium, and folate, and (ii) an exercise-sleep-social pattern marked by weekly physical activities like bowling, bicycling, sleep quality (low snoring/sleepiness), and high social engagement. Neither pattern was associated with multimorbidity cross-sectionally. Over a median 5.8-year follow-up (n = 869; 140 deaths), participants in the upper tertiles for combined lifestyle pattern scores had a 20% lower mortality risk than those in the lowest tertile [adjusted HR: 0.80 (95% CI: 0.65–0.97); *p*-trend = 0.02]. This association was stronger in participants with multimorbidity, with a 29% lower risk [0.71 (0.56–0.89); *p*-trend = 0.01], likely due to multimorbidity modifying the relationship between nutrition and mortality risk (*p*-interaction < 0.05). While multimorbidity did not modify the relationship between the exercise-sleep-social pattern and risk of mortality, it was consistently associated with a 19–20% lower risk (*p*-trend < 0.03), regardless of the multimorbidity status. **Conclusions:** Older adults with multimorbidity may particularly benefit from adopting healthy lifestyles focusing on nutrition, physical activity, sleep quality, and social engagement to reduce their mortality risk.

## 1. Introduction

Multimorbidity, defined as the presence of two or more chronic conditions, affects over half of adults aged 60 years and above, contributing to increased risks of mortality, disability, adverse drug events, and reduced quality of life [1,2,3]. Despite these challenges, current clinical guidelines predominantly address individual diseases rather than multimorbidity. This fragmented approach often leads to polypharmacy (the concurrent use of five or more medications simultaneously [4]), which may result in overtreatment and an increased risk of medication-related complications, particularly in older adults [5,6]. In contrast, lifestyle medicine presents a holistic, patient-centred, and cost-effective strategy for managing multimorbidity [3,7], potentially mitigating the risks associated with medication overload [8,9].

Healthy lifestyle behaviours, including diet, physical activity, and sleep, are well-established as key factors in disease prevention and management in older adults [10]. While previous studies have shown that individual lifestyle factors may reduce the risk of multimorbidity and increase life expectancy [11,12], the combined effect of multiple lifestyle behaviours is likely greater than the sum of its parts due to their synergistic interactions. A composite lifestyle score offers a more integrative perspective by capturing these interconnections; however, the definitions and components of lifestyle indices vary across studies, making direct comparisons challenging [11,12,13].

For instance, the UK Biobank Study included physical activity, smoking, diet (fruit and vegetable consumption only), and alcohol use [11], while the English Longitudinal Study of Ageing additionally incorporated body mass index (BMI) [12]. The Women’s Health Initiative used a guideline-based composite index comprising smoking, diet quality, alcohol use, BMI, physical activity, and sleep-based activity [13]. These discrepancies highlight the lack of a standardised lifestyle index, limiting the generalisability of the findings across different populations.

To address this methodological inconsistency, we employed a data-driven approach, principal component analysis (PCA), to identify dominant lifestyle patterns among older adults based on key lifestyle factors, including nutrition, physical activity, smoking, alcohol use, sleep quality, and social engagement. The inclusion of social engagement is a novel aspect of this study, as it has been independently associated with mortality [14], depression [15,16], dementia [17,18], and overall well-being [14,16] in older adults. While previous studies have primarily focused on conventional lifestyle factors, integrating social engagement provides a more comprehensive assessment of lifestyle patterns relevant to healthy ageing.

Furthermore, recognising the potential limitations of a data-driven approach, we conducted sensitivity analyses using a guidelines-based lifestyle index (including diet quality, cigarette smoking, alcohol use, and physical activity according to the Australian health guidelines) to validate the findings of our data-driven approach. This lifestyle index was developed in the same cohort as that of the current study [19].

This dual approach strengthens the robustness of our findings. By integrating a comprehensive, multidimensional lifestyle assessment and employing both data-driven and guideline-based approaches, this study is the first to provide critical insights into how lifestyle behaviours interact with multimorbidity to influence mortality risk in older adults.

Hence, this study aimed to assess the association between data-driven lifestyle patterns and multimorbidity and mortality risk in a cohort of older Australian adults. We explored whether multimorbidity at baseline modified or mediated the relationship between a lifestyle pattern and mortality risk over time. We hypothesised that adherence to a healthy lifestyle, either in a data-driven or guideline-based approach, would be associated with lower mortality risk, regardless of multimorbidity status at baseline.

## 2. Methods

### 2.1. Study Population

The Sydney Memory and Ageing Study (MAS), a population-based longitudinal cohort study, was designed to investigate ageing and cognition. A sample of 1037 Australians aged 70–90 years was recruited between 2005 and 2007 from the Sydney Eastern suburbs living in the community without a current diagnosis of dementia. Participants underwent biennial in-person assessments comprising cognitive and/or neuropsychological tests, medical assessments, and survey questionnaires on sociodemographic, health status, and lifestyle factors. A close friend or family member was also interviewed to provide further information. Participants were followed up from baseline until the fourth follow-up assessment for mortality from 2011 to 2013. The details of the cohort protocol, including the inclusion and exclusion criteria and descriptive statistics, have been published previously [20]. Briefly, participants were required to have sufficient English proficiency to complete the psychometric assessment. Over 90% of the participants had an informant, who needed to be the closest person and, ideally, a co-habitant to report on the participant’s memory, thinking, and daily functions. Participants with neurological or psychiatric conditions, severe medical conditions, cognitive impairment, or diagnosed dementia were deemed ineligible to participate in the study.

This study was approved by the South Eastern Sydney Area Health Service Human Research Ethics Committee and the University of New South Wales Human Research Ethics Committee (Approval Code: HREC 05037). All participants and their informants provided informed consent to participate in the cohort.

### 2.2. Baseline Assessment

At the baseline assessment, demographics (age, sex, years of education, and English-speaking background), history of health conditions (metabolic health diseases, respiratory conditions, cancers, musculoskeletal disorders, neurological disorders, among others); lifestyle factors (habitual diets, physical activities, sleep measurement, tobacco smoking, alcohol use, and social engagement activities) were surveyed among 1037 participants.

Habitual diet was estimated using the Dietary Questionnaire for Epidemiological Studies Version 2 (DQES v2), an 80-item validated Food Frequency Questionnaire (FFQ) developed by the Cancer Council of Victoria, including seventy-four food items and six alcoholic beverages [21,22,23]. Nutrient intake estimates were calculated by the Cancer Epidemiology Centre of the Cancer Council in Victoria using the Australian food composition NUTTAB database [24]. Valid total energy intake varied from 1857.3 to 15,225.3 kilojoules among 957 participants at baseline.

Physical activity assessment was undertaken using self-report questionnaires to measure the duration (minutes/week) of the following activities: bowling, golfing, tennis, swimming, dancing, bicycling, and aerobics. For sleep, two questions were asked to estimate sleep quality, “Snore: have you been told you snore?” and “Sleepiness: do you have excessive sleepiness during the day that interferes with normal functioning?” For social engagement levels, two questions were used at baseline, including “Do you have someone you can confide in?” and “What is the number of face-to-face contacts per month?”

Body weight and height were measured or self-reported at baseline. BMI was calculated as weight (kg)/height (m^2^). Other measures included basic activities of daily living (ADL) based on Lawton and Brody (1969) [25], including ambulating, feeding, dressing, bathing, grooming, and continence (bladder and bowel functions). The total ADL score ranged from 7 to 35, with the lowest scores reflecting maximal independence and the highest scores reflecting maximal dependence.

Multimorbidity status was defined as the presence of at least two of the most common disease burdens in Australia [26], including arthritis, asthma, cancer, chronic kidney disease, chronic obstructive pulmonary disease (COPD), diabetes, cardiovascular diseases, depression, and osteoporosis. Although back pain is listed among the AIHW’s common disease burdens, we could not include it in the definition of multimorbidity due to its absence in the cohort data collection. Based on each of the self-reported physician diagnoses at baseline, relevant conditions were grouped into each of the nine disease burdens. These conditions included acute myocardial infarction, angina, aortic aneurysm, arthritis (osteoarthritis, rheumatoid arthritis, gout, and others), asthma, atrial fibrillation, cancer, cardiomyopathy, chronic kidney disease, COPD, depression, diabetes, heart valve disease, osteoporosis, stroke, and transient ischaemic attack.

A comorbidity index was developed based on the disease weights contributing to mortality risk two years after baseline in a Cox proportional hazards model, a common method for estimating the severity of disease [27]. For the nine groups of disease burden, based on the hazard ratios for mortality risk, asthma, arthritis, cancer, and diabetes were assigned a weight of 1; chronic kidney disease, osteoporosis including fractures, depression, and cardiovascular diseases were assigned a weight of 2; and COPD was assigned a weight of 6. Hence, the comorbidity index in the analytical sample ranged from 0 (no comorbidity) to 14 (maximal comorbidity), with a median of 3 (interquartile range, 2–4). Multimorbidity was defined as the presence of two or more disease groups [28]. The correlation coefficient between the comorbidity index and multimorbidity based on counts was 0.70 (*p* < 0.05). Hence, they were not simultaneously adjusted in the model.

### 2.3. Exposures of Interest

Lifestyle patterns were derived based on the baseline nutrient intake from habitual diet estimation, physical activities, cigarette smoking status, alcohol use, sleep behaviours, and social engagement activities mentioned above using principal component analysis (PCA) conducted among those with complete data on these nutrition and lifestyle variables (n = 895). The PCA procedure began by including 28 vitamins and minerals, various physical activities, sleep quality variables, cigarette smoking status, alcohol use status, and two social engagement variables.

After multiple PCA procedures and based on the criteria, including eigenvalues (≥1.3), component loadings (>0.30) in varimax rotation, sampling adequacy (>0.8), and interpretability, two patterns were identified: the first pattern included dietary intake of protein, fibre, iron, zinc, magnesium, potassium, and folate, which we named a nutrition pattern; the second pattern included bowling, bicycling, low likelihood of snoring, low sleepiness, having a confidant, and maintaining monthly face-to-face contact, which we named an exercise-sleep-social pattern.

The criteria for PCA cut-offs (eigenvalues ≥ 1.3 and factor loadings > 0.30) were chosen to ensure that the identified principal components (patterns) meaningfully represented the underlying lifestyle patterns in the study population. While selecting components with eigenvalues ≥ 1 is a common criterion based on Kaiser’s rule [29], six principal components (patterns) were derived with eigenvalues greater than 1 in the PCA analysis. Hence, we retained the two most prominent components (patterns) with eigenvalues greater than 1.3 to represent meaningful and interpretable lifestyle patterns. A factor loading of 0.30 is widely accepted in epidemiological and behavioural studies as the minimum threshold for meaningful variable inclusion [30], which indicates moderate correlations among the identified variables. This threshold also ensured that the identified patterns were driven by meaningful correlations rather than weak associations.

The two patterns were distinct, with a correlation coefficient of 0.057, a scale reliability coefficient of 0.68, and a Kaiser-Meyer-Olkin measure of 0.85, indicating sufficient sampling adequacy. The two distinct lifestyle patterns explained 47% of the total variance in the lifestyle variables included. The lifestyle pattern scores were linear variables and were computed as the sum of the standardised frequencies of each lifestyle variable associated with each pattern, with zero as the mean. We divided the two pattern scores into tertiles based on the distribution of the overall cohort, with the low tertile (tertile 1) representing the least healthy lifestyle pattern and the high tertile (tertile 3) representing the healthiest lifestyle pattern. Supplementary Table S1 describes the variables included in each pattern.

### 2.4. Main Outcomes

Deaths were ascertained through the National Death Index; where this information was unavailable at the end of wave 4 (2011–2013), we relied upon information from Ryerson Obituaries, relatives or friends, and hospital/nursing home staff to confirm participants’ death status, age at death, and date of death. In this analytic sample, 14 people were lost to follow-up, and 12 were not assessed or withdrew from the study during follow-up. There were 140 deaths until the end of wave 4 (2011–2013), the censored period for this analysis.

### 2.5. Statistical Analysis

For each study participant, person-years were counted from the date of the baseline interview (2005–2007) to the date of death, withdrawal from the study, no assessment, or the last date of follow-up (31 December 2013), whichever occurred first.

Baseline characteristics and lifestyle factors or health indicators were described across the tertiles of each lifestyle pattern score, including age, sex, language background, level of education, BMI, cigarette smoking, alcohol use, total energy intake, basic ADL scores, and comorbidity index.

#### 2.5.1. Cross-Sectional Analysis

To assess the association between a lifestyle pattern and the prevalence of multimorbidity at baseline, multivariable logistic regression models were used to estimate the odds ratios (ORs) and 95% confidence intervals (CIs) to compare a healthier lifestyle pattern [i.e., mid tertile (tertile 2) or high tertile (tertile 3)] with the low tertile, the least healthy pattern (tertile 1).

#### 2.5.2. Longitudinal Analysis

To assess mortality risk among those with multimorbidity at baseline, the Cox proportional hazards model was applied to assess the association between a healthier lifestyle pattern (mid or high tertile) and the least healthy lifestyle pattern (low tertile) for the risk of death to estimate hazard ratios (HRs) and 95% CIs. The proportional assumption was tested using scaled Schoenfeld residuals and a specified function of time. No violations were detected for any of the variables in the Cox models, except for the third tertile of the exercise-sleep-social pattern score. Therefore, the analysis included a time-varying function for the exercise-sleep-social pattern tertiles in all Cox models.

To assess whether the nutrition and exercise-sleep-social patterns synergistically contributed to the odds of having multimorbidity cross-sectionally or the relative risk of mortality longitudinally, we created an ordinal variable combining the lowest tertile scores of both patterns to divide the cohort into four groups: the referent group included those in the low-tertiles of both patterns, representing the least healthy lifestyle; the less healthy groups included the low tertile of one pattern with the upper tertiles of the other pattern (i.e., tertile1 of nutrition or exercise-sleep-social pattern with tertiles 2–3 of the other pattern), representing those either eat healthily but less active or those active but eat unhealthily; the healthiest group included those in the higher tertiles of both patterns (i.e., mid- and high-tertiles of both patterns), representing those adhering to healthy eating and being active. This grouping method yielded four groups, which provided a sufficient sample size (rather than nine groups) for analysis.

For prevalent multimorbidity at baseline in the cross-sectional analysis, the models included age (years), sex (men, women), English-speaking background (English, non-English), level of education (no formal education, primary school, secondary school or higher), BMI (kg/m^2^), cigarette smoking (never, former, current), alcohol use (never, former, current), total energy intake (kilojoules/day), and basic ADL scores.

For the longitudinal analysis of mortality, the models included the above-mentioned covariates and the comorbidity index.

To examine a linear trend, the median value of the tertiles of the lifestyle pattern score was entered as a continuous variable in the regression model to assess dose-response relationships.

To assess the mediating role of multimorbidity, the primary criteria that needed to be met included statistically significant relationships between a lifestyle pattern and multimorbidity and between multimorbidity and mortality risk. For effect modification, if the interaction term, including a nutrition or exercise-sleep-social pattern and multimorbidity in the regression model, is statistically significant in the association with mortality risk, we will conduct a stratified analysis among those with multimorbidity (n = 596) at baseline.

A lifestyle index, including diet, physical activity, alcohol use, and cigarette smoking [19], which was based on several Australian guidelines on diet, physical activity, alcohol use, and tobacco smoking, was included in the sensitivity analysis to assess the accordance of the results yielded from the data-driven lifestyle patterns in descriptive and regression analyses. By integrating a comprehensive, multidimensional lifestyle assessment and employing both data-driven and guidelines-based approaches, this lifestyle index was divided as having 0 (none), 1, 2, 3, or 4 risk factor(s) if meeting the following criteria (Supplementary Table S2): poor diet: participants in the lowest tertile of the dietary score based on the Australian Dietary Guidelines [31]; physical inactivity: less than 150 min of moderate-to-vigorous intensity activity weekly per the Australian and international guidelines [32]; alcohol use: over ten standard drinks per week [33]; and current smoking [33]. The covariates included in the regression models for the cross-sectional analysis were age, sex, education level, body mass index (kg/m^2^), activities of daily living, total energy intake, language background, snoring, sleepiness, and having a confidant. The longitudinal regression models adjusted for the above plus a comorbidity index.

All statistical analyses were performed using Stata 18 (Stata Corporation, College Station, TX, USA). The PCA procedures were conducted using the “pca” command in Stata. All reported *p*-values were 2-sided, and a value less than 0.05 was considered statistically significant.

## 3. Results

**Figure 1:**
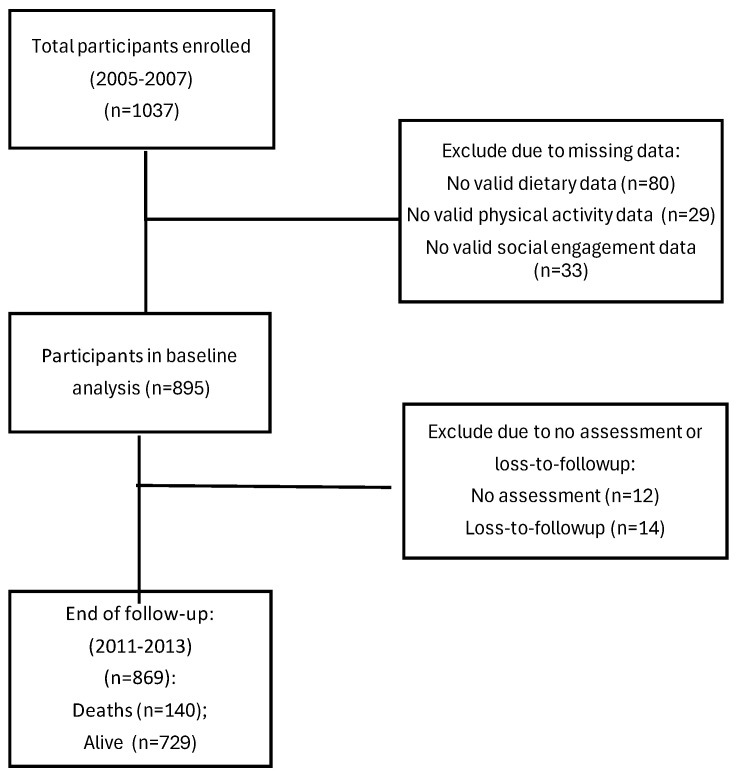
Flow chart of the included participants in the Sydney Memory and Ageing Study from enrolment at baseline (2005–2007) to wave 4 (2011–2013).

Among the 1037 participants at baseline, 895 were included in the analysis, with a mean age of 78.2 (SD: 4.8) years and 56.3% females. During a median follow-up of 5.8 (interquartile range: 4.0, 5.9) years (n = 869), 140 deaths were recorded, 14 participants were lost to follow-up, and 12 participants were not assessed (Figure 1).

The demographic characteristics of each lifestyle pattern tertile score are described in Table 1. The distributions of age and education were similar across each pattern of tertile scores. Men tended to have higher nutrition pattern scores but lower exercise-sleep-social pattern scores than women did. The distributions of BMI, smoking, and alcohol use did not vary significantly for each tertile score. The (basic) ADL scores were around 7, which was similar across the pattern scores by tertile, indicating independence in daily living activities. Regarding the comorbidity index, the upper 25% percentile in the low tertile of the nutrition pattern had the highest score of 5, and the high tertile of the exercise-sleep-social score had the lowest score of 1 in the 25th percentile. Finally, over two-thirds of the study sample had multimorbidity.

In the cross-sectional analysis for multimorbidity at baseline, no statistically significant association was found for each lifestyle pattern or the combined patterns with mortality, even though the risk estimates suggested a lower risk by comparing the highest tertile with the lowest tertile score (Table 2). Because of the non-significant results, mediation analysis was not performed.

In the longitudinal analysis for mortality risk, there was an inverse relationship, i.e., a higher tertile lifestyle pattern score was associated with a lower mortality risk, in which a significant result was observed in the high tertile of the exercise-sleep-social pattern score compared to the lowest tertile, yielding a 17% lower risk (HR: 0.83; 95% CI: 0.71, 0.98), with a significant trend across the tertiles (*p* for trend = 0.025). Combining the two patterns, we found a significant trend between higher tertile scores (mid- and high-tertiles) and lower mortality risk (*p* for trend = 0.02). Participants in the upper tertiles of the nutrition and exercise-sleep-social pattern scores had a 20% lower mortality risk (HR: 0.80; 95% CI: 0.65–0.97) than those in the lowest tertile scores. The association between the nutrition pattern and mortality risk was not statistically significant, although it trended toward a lower mortality risk in higher tertile scores (Table 3).

Furthermore, we identified that multimorbidity status modified the association between the nutrition pattern and mortality risk (*p* < 0.05). Due to the study sample size, we only analysed the data of those with multimorbidity at baseline (67% of the study sample). In this sub-group analysis, the nutrition pattern was not associated with mortality risk. However, the mid versus low tertile association was borderline significant (*p* = 0.08), with a 39% lower risk (HR: 0.61; 95% CI: 0.34, 1.07). In contrast, a higher exercise-sleep-social pattern score (high versus low tertile) was significantly associated with a 19% lower mortality risk (HR: 0.81; 95% CI: 0.67, 0.98), with a significant linear trend (*p* for trend = 0.03). Combining the two lifestyle patterns, we found a significant trend indicating that a healthy lifestyle was associated with lower mortality risk (*p* for trend = 0.01), with a hazard ratio of 0.71 (95% CI: 0.56, 0.89) in the higher tertiles of the pattern scores, i.e., a 29% lower risk of mortality (Table 4).

The sensitivity analysis using the lifestyle index based on Australian health guidelines suggests a significant trend (*p* = 0.02) associated with multimorbidity at baseline. Significant results were observed among those with two or fewer risk factors compared to those with three or four (Table 2). However, the results for mortality risk were not statistically significant, although the results suggest that the fewer the risk factors, the lower the mortality risk (Table 3 and Table 4).

## 4. Discussion

**Figure 2:**
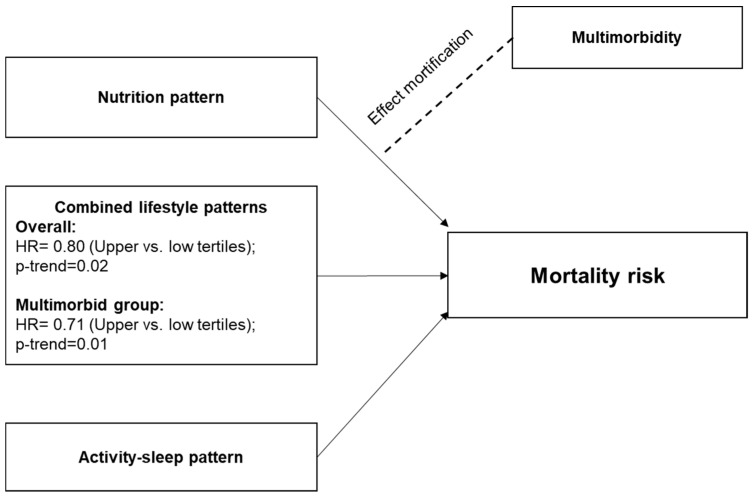
Summary of findings.

This study employed an empirical, data-driven approach to identify two primary lifestyle patterns in a cohort of older Australians, a nutrition pattern and an exercise-sleep-social pattern, and evaluated their associations with multimorbidity prevalence and mortality risk. We found that a healthy lifestyle combining the upper tertiles of nutrition and exercise-sleep-social patterns significantly reduced mortality risk by 20–29%, with a significant trend for the overall cohort and those with multimorbidity (Figure 2). Specifically, adherence to higher tertile scores of the exercise-sleep-social pattern alone was associated with a lower mortality risk, regardless of multimorbidity status. Additionally, those with better nutrition tended to be less prone to mortality than those with multimorbidity. These results highlight the significant role of adopting a healthy lifestyle, including diet and nutrition, physical activity, sleep quality, and social engagement, in managing multimorbidity in older adults.

Using principal component analysis to derive lifestyle patterns, we found results consistent with those using a lifestyle score or lifestyle index generated based on different health guideline recommendations, such as those related to the prevention of heart disease or cancer and lifestyle recommendations related to diet and nutrition, physical activity, tobacco, and alcohol use [11,12,13]. Furthermore, our results are consistent with those of other studies assessing lifestyle patterns associated with mortality risk [34,35].

The most notable finding of this study was that nutrition appeared to play a more critical role in lowering mortality risk among participants with multimorbidity at baseline, as indicated by the significant interaction term between the nutrition pattern and multimorbidity. While the nutrition pattern alone was not significantly associated with mortality risk (likely due to the lack of statistical power to detect differences between the tertiles), when combined with high exercise-sleep-social scores, a stronger protective association was evident among those with multimorbidity, we observed a 29% (HR: 0.71; 95% CI: 0.59, 0.89) risk reduction compared with a 20% risk reduction among those in the overall sample (HR: 0.80; 95% CI: 0.65, 0.97). This suggests that the nutrition pattern may have a stronger effect on mortality among those with multimorbidity.

Consistent with our findings, a Japanese study also suggested that 4–8 more life years were gained in those aged 50–80 years with multimorbidity who adopted at least six healthy lifestyle factors compared with those who adopted 0–2 healthy lifestyles [36]. Eight healthy lifestyle factors were included in the Japanese cohort: daily fruit, fish, and milk consumption; habitual exercise or walking; BMI between 21 and 25 kg/m^2^; alcohol intake equivalent to less than 46 g/day ethanol; never smoking; and sleep duration of 5.5–7.4 h/day. Interestingly, those meeting at least six of the factors were 80–90% more likely to meet the dietary requirements, indicating the significant health benefits of food and nutrition contributing to life years gained in people with multimorbidity [36].

Our study, a Japanese study [36], and the UK Biobank Study [11] found that greater adherence to a healthy lifestyle was consistently associated with a lower risk of mortality or increased life years gained. Although this association was stronger among individuals with multimorbidity in our study and the Japanese study, the UK Biobank Study found similar associations regardless of multimorbidity. This difference may be due to the younger age group in the UK Biobank Study, which had a median age of 58 years (range 38–73) [11].

Consistently, our findings indicate an inverse association between higher exercise-sleep-social pattern scores and lower mortality risk in the overall sample and among individuals with multimorbidity. This suggests that combined physical activity, sleep quality, and social engagement play crucial roles in a longer lifespan, regardless of multimorbidity status.

To our knowledge, no study has examined the combined impact of physical activity, sleep quality, and social engagement on mortality risk in individuals with multimorbidity. However, each of these factors has been studied separately. In the UK Biobank Study, physical activity showed an inverse dose-response relationship with mortality risk, with gains in life expectancy regardless of the multimorbidity status [11]. Similarly, in the Survey of Health, Ageing, and Retirement in Europe (SHARE), physical activity was associated with better self-rated health, muscle strength, and well-being, although these benefits diminished after the age of 70 years [37]. Both studies highlight the health benefits for older age groups, irrespective of multimorbidity.

Sleep quality appears to play a moderate role in mortality risk. In the UK Biobank, a 1-point increase in sleep score (factors including early chronotype, 7–8 h of sleep, and no insomnia) reduced mortality risk by 10% [38], while sleeping less than five hours per night increased the multimorbidity risk by 30% [39]. However, no association with mortality was found in patients with multimorbidity. Similarly, in the Canadian Longitudinal Study on Aging, neither sleep duration nor sleep satisfaction significantly influenced multimorbidity risk in those 75 years and older [40]. These studies suggest that the role of sleep quality in multimorbidity and mortality risk remains unclear [41].

Social engagement and mortality risk are interconnected through complex mechanisms. Individuals with better physical and cognitive functions tend to engage more socially, gaining psychological and physical health benefits [14]. Conversely, those with multiple chronic conditions experience a decline in social engagement [42,43]. Given the high prevalence of multimorbidity in adults over 70 years of age, practical strategies to enhance social engagement are crucial for improving well-being and reducing adverse health outcomes.

Existing research, including our study, collectively underscores the benefits of physical activity, sleep quality, and social engagement on overall well-being. Because maintaining physical activity and social engagement is challenging for older adults, this possibly explains why multimorbidity did not modify the association between the exercise-sleep-social pattern and mortality risk in our findings.

Our sensitivity analyses agreed with studies using a lifestyle index defined by diet quality, physical activity, smoking, and drinking based on health guidelines [11,12,13]. We observed significant trends and results associated with multimorbidity at the baseline. Although the results for mortality risk were not statistically significant using the lifestyle index, the results were as expected. The non-significant results for mortality risk are likely due to the lack of statistical power to detect differences, as there were far fewer deaths in those with a low number of risk factors, with only five cases in the category of zero risk factors and 29 in the category of three to four risk factors.

To our knowledge, this is the first study to identify the role of lifestyle patterns in mortality among older adults with multimorbidity using a dual methodology to define lifestyle patterns. A strength of this study is that it incorporated several nuanced lifestyle factors, including sleep quality and social engagement, along with nutrition and physical activity, to generate comprehensive and distinct lifestyle patterns. Although the derived nutrition and exercise-sleep-social patterns were empirically driven in this study, their results as protective measures against mortality risk were aligned with studies using lifestyle indices based on health guideline recommendations.

Our study also has limitations. Self-reported lifestyle factors are prone to recall and response biases and potentially result in misclassification, even though this will likely be non-differential, leading to underestimating the observed associations with the health outcomes. Additionally, sleep quality was not assessed using a validated sleep questionnaire in this cohort study. Instead, we estimated sleep quality based on two relevant questions regarding snoring and excessive daytime sleepiness. As a result, the overall sleep quality of the participants may not have been fully captured. To mitigate these biases, we conducted a sensitivity analysis using an alternative approach to estimate lifestyle indexes, allowing us to compare the results from both methods and enhance the robustness of our findings. While we defined multimorbidity based on common disease burdens in Australia, the estimated prevalence among adults aged 70–90 years aligns with the existing literature [2,4]. However, self-reported physician-diagnosed health histories may be subject to recall bias. One way to address this could be to randomly select a subset of participants to verify their self-reported diagnoses against physicians’ health records to assess their accuracy. Additionally, the absence of medication data could introduce residual confounding, potentially biasing the associations assessed. As with all observational studies, self-reported survey data are an inherent limitation, and it is difficult to eliminate potential biases entirely. Hence, our findings do not establish causality, and residual confounding remains a possibility despite our mitigation efforts. Finally, the generalisability of our findings may be limited, as the study population was relatively homogeneous, primarily metropolitan residents, highly educated, and predominantly English-speaking older adults in Australia.

## 5. Conclusions

Our findings in a cohort of Australian adults aged 70–90 years highlight the significant health benefits of adopting a healthy lifestyle, including balanced nutrition, regular physical activity, quality sleep, and active social engagement, in reducing mortality risk. Notably, diet and nutrition play important roles in improving health outcomes, particularly in patients with multimorbidity.

These results emphasise the importance of routinely monitoring and supporting key lifestyle factors in older adults to promote healthy ageing. Strategies such as preventing malnutrition, encouraging low-impact exercise, promoting good sleep hygiene, and fostering social connections can help maintain health and improve the quality of life in both self-care and clinical practice. Integrating these habits into daily routines and healthcare settings may be especially beneficial for older adults with multiple chronic conditions.

## Figures and Tables

**Table 1:** Baseline characteristics according to the tertile scores of the nutrition pattern and the exercise-sleep-social pattern (n = 895).

	Nutrition Pattern			Exercise-Sleep-Social Pattern			
	Tertile 1	Tertile 2	Tertile 3	Tertile 1	Tertile 2 (Middle)	Tertile 3	Total
	(Low)	(Middle)	(High)	(Low)		(High)	
**N**	299 (33.4%)	298 (33.3%)	298 (33.3%)	299 (33.4%)	298 (33.3%)	298 (33.3%)	895 (100.0%)
Age (years) **	78.4 (4.9)	78.4 (4.9)	77.9 (4.6)	78.4 (4.7)	78.1 (4.9)	78.2 (4.8)	78.2 (4.8)
**Sex**							
Men	81 (27.1%)	127 (42.6%)	183 (61.4%)	168 (56.2%)	114 (38.3%)	109 (36.6%)	391 (43.7%)
Women	218 (72.9%)	171 (57.4%)	115 (38.6%)	131 (43.8%)	184 (61.7%)	189 (63.4%)	504 (56.3%)
**Education**							
High school or below	207 (69.2%)	219 (73.5%)	198 (66.4%)	206 (68.9%)	216 (72.5%)	202 (67.8%)	624 (69.7%)
Associate degree	29 (9.7%)	26 (8.7%)	25 (8.4%)	25 (8.4%)	24 (8.1%)	31 (10.4%)	80 (8.9%)
University or above	63 (21.1%)	53 (17.8%)	75 (25.2%)	68 (22.7%)	58 (19.5%)	65 (21.8%)	191 (21.3%)
**Language background**							
English-speaking	258 (86.3%)	252 (84.6%)	257 (86.2%)	255 (85.3%)	249 (83.6%)	263 (88.3%)	767 (85.7%)
Non-English-speaking	41 (13.7%)	46 (15.4%)	41 (13.8%)	44 (14.7%)	49 (16.4%)	35 (11.7%)	128 (14.3%)
**Body mass index (kg/m^2^)**	26.7 (4.4)	27.4 (4.7)	27.3 (4.4)	28.0 (4.9)	27.2 (4.6)	26.2 (3.8)	27.1 (4.5)
**Smoking status**							
Never	158 (53.0%)	139 (46.6%)	119 (39.9%)	121 (40.6%)	130 (43.6%)	165 (55.4%)	416 (46.5%)
Former	129 (43.3%)	148 (49.7%)	167 (56.0%)	163 (54.7%)	153 (51.3%)	128 (43.0%)	444 (49.7%)
Current	10 (3.4%)	9 (3.0%)	9 (3.0%)	13 (4.4%)	11 (3.7%)	4 (1.3%)	28 (3.1%)
Missing	1 (0.3%)	2 (0.7%)	3 (1.0%)	1 (0.3%)	4 (1.3%)	1 (0.3%)	6 (0.7%)
**Alcohol use**							
Never	21 (7.0%)	17 (5.7%)	11 (3.7%)	23 (7.7%)	15 (5.0%)	11 (3.7%)	49 (5.5%)
Past	20 (6.7%)	20 (6.7%)	18 (6.0%)	16 (5.4%)	20 (6.7%)	22 (7.4%)	58 (6.5%)
Current	258 (86.3%)	261 (87.6%)	269 (90.3%)	260 (87.0%)	263 (88.3%)	265 (88.9%)	788 (88.0%)
Energy intake (kJ/day) **	4489.3 (884.3)	6123.3 (841.7)	8580.3 (1692.9)	6814.3 (2190.9)	6114.3 (1980.7)	6244.7 (1961.0)	6390.6 (2066.8)
**Basic Activities of Daily Living** (ADL) [**7 (maximal independence)-35 (maximal dependence)**]	7.3 (1.1)	7.1 (0.7)	7.2 (0.6)	7.3 (1.2)	7.1 (0.7)	7.1 (0.4)	7.2 (0.8)
**Comorbidity index (median, interquartile range)**	3 (2, 5)	3 (2, 4)	3 (2, 4)	3 (2, 5)	3 (2, 4)	3 (1, 4)	3 (2, 4)
**Multiple conditions (multimorbidity) at baseline**							
None	31 (10.4%)	29 (9.7%)	31 (10.4%)	33 (11.0%)	26 (8.7%)	32 (10.7%)	91 (10.2%)
One condition	64 (21.4%)	77 (25.8%)	66 (22.1%)	58 (19.4%)	67 (22.5%)	82 (27.5%)	207 (23.1%)
Two or more conditions	204 (68.2%)	192 (64.4%)	201 (67.4%)	208 (69.6%)	205 (68.8%)	184 (61.7%)	597 (66.7%)
Lifestyle index based on number of risk factors * **(n = 877)**							
3–4 risk factors	64 (49.0%)	41 (31.8%)	24 (18.6%)	57 (44.2%)	48 (37.2%)	24 (18.6%)	61 (7.0%)
2 risk factors	116 (39.5%)	96 (32.7%)	82 (27.9%)	125 (42.5%)	86 (29.3%)	83 (28.2%)	393 (44.8%)
1 risk factor	96 (24.4%)	141 (35.9%)	156 (39.7%)	102 (26.0%)	143 (36.4%)	148 (37.7%)	294 (33.5%)
None	14 (23.0%)	18 (29.5%)	29 (47.5%)	9 (14.8%)	14 (23.0%)	38 (62.3%)	129 (14.7%)

* Risk factors are estimated for diet, physical activity, smoking, and alcohol use. ** Values are in mean (standard deviation) if not specified.

**Table 2:** Lifestyle patterns in association with multimorbidity cross-sectionally (n = 895).

Lifestyle Patterns or Individual Factors	Multimorbidity (%)	Odds Ratio	95% Confidence Interval	
Nutrition pattern ^1^				
Tertile 1 (n = 299)	68.2	Ref		
Tertile 2 (n = 298)	64.4	0.80	0.53	1.22
Tertile 3 (n = 298)	67.5	0.82	0.43	1.55
*p* for trend		0.53		
Exercise-sleep-social pattern ^1^				
Tertile 1 (n = 299)	69.6	Ref		
Tertile 2 (n = 298)	68.6	1.09	0.75	1.59
Tertile 3 (n = 298)	61.7	0.83	0.57	1.21
*p* for trend		0.36		
Combined nutrition with exercise-sleep-social patterns ^1^				
Tertile 1 for both (n = 91)	71.4	Ref		
Nutrition tertile 2–3 and activity tertile 1 (n = 208)	68.8	0.73	0.38	1.39
Nutrition tertile 1 and activity tertile 2–3 (n = 208)	66.8	0.86	0.48	1.54
Nutrition tertile 2–3 and activity tertile 2–3 (n = 388)	64.4	0.72	0.40	1.27
*p* for trend		0.23		
Sensitivity analysis: based on the number of risk factors * ^2^				
3–4 risk factors (n = 107)	76.4	Ref		
2 risk factors (n = 323)	68.4	0.70	0.42	1.15
1 risk factor (n = 420)	64.8	0.61	0.38	1.00
None (n = 66)	51.5	0.35	0.18	0.70
*p* for trend		0.02		

* Risk factors are estimated for diet, physical activity, smoking, and alcohol use. ^1^ Model adjusted for age (years), sex (men, women), education level (high school, associate degree, and university), body mass index (kg/m^2^), smoking status (never, former, and current), alcohol use (never, former, and current), activities of daily living (7–35), total energy intake (kilojoule), and language background (English, non-English). ^2^ Models were adjusted for age (years), sex (men, women), education level (high school, associate degree, and university), body mass index (kg/m^2^), activities of daily living (7–35), total energy intake (kilojoule), language background (English, non-English), snoring (rarely, occasionally, regularly), sleepiness (rarely, slightly dozing, heavy sleepiness), and having a confidant (yes, no).

**Table 3:** Lifestyle patterns in association with overall mortality (n = 869).

Lifestyle Patterns	Person-Years (PY)	Death	Hazard Ratio	95% Confidence Interval	
Nutrition pattern ^1^					
Tertile 1	1470.1	51	Ref		
Tertile 2	1430.7	42	0.71	0.43	1.16
Tertile 3	1477.8	47	0.67	0.32	1.42
*p* for trend			0.30		
Exercise-sleep-social pattern ^1^					
Tertile 1	1427.6	65	Ref		
Tertile 2	1445.4	40	0.91	0.79	1.05
Tertile 3	1505.5	35	0.83	0.71	0.98
*p* for trend			0.025		
Combined nutrition with exercise-sleep-social patterns ^1^					
Tertile 1 for both patterns	435.9	20.0	Ref		
Nutrition tertile 2–3 and activity tertile 1	991.7	45.0	0.97	0.79	1.20
Nutrition tertile 1 and activity tertile 2–3	1034.1	31.0	0.96	0.79	1.17
Nutrition tertile 2–3 and activity tertile 2–3	1916.8	44.0	0.80	0.65	0.97
*p* for trend			0.02		
Lifestyle index based on number of risk factors * ^2^					
3–4 risk factors	618.8	29.0	Ref		
2 risk factors	1437.2	46.0	0.65	0.39	1.08
1 risk factor	1905.1	60.0	0.78	0.47	1.28
None	323.7	5.0	0.37	0.11	1.24
*p* for trend			0.28		

* Risk factors are estimated for diet, physical activity, smoking, and alcohol use. ^1^ Models were adjusted for age (years), sex (men, women), education level (high school, associate degree, and university), body mass index (kg/m^2^), smoking status (never, former, and current), alcohol use (never, former, and current), activities of daily living (7–35), total energy intake (kilojoule), language background (English, non-English), and comorbidity index. ^2^ Models were adjusted for age (years), sex (men, women), education level (high school, associate degree, and university), body mass index (kg/m^2^), activities of daily living (7–35), total energy intake (kilojoule), language background (English, non-English), snoring (rarely, occasionally, regularly), sleepiness (rarely, slightly dozing, heavy sleepiness), having a confidant (yes, no), and comorbidity index.

**Table 4:** Lifestyle patterns in association with mortality among those with multimorbidity (n = 597).

Lifestyle Patterns	Person-Years (PY)	Death	Hazard Ratio	95% Confidence Interval	
Nutrition pattern					
Tertile 1	988.5	47.0	Ref		
Tertile 2	908.9	29.0	0.61	0.34	1.07
Tertile 3	973.6	35.0	0.60	0.25	1.40
*p* for trend			0.20		
Exercise-sleep-social pattern					
Tertile 1	968.1	52.0	Ref		
Tertile 2	978.7	35.0	0.92	0.78	1.07
Tertile 3	924.2	24.0	0.81	0.67	0.98
*p* for trend			0.03		
Combined nutrition with exercise-sleep-social patterns ^1^					
Tertile 1 for both	303.8	18.0	Ref		
Nutrition tertile 2–3 and activity tertile 1	664.4	34.0	0.93	0.74	1.17
Nutrition tertile 1 and activity tertile 2–3	684.8	29.0	1.01	0.82	1.23
Nutrition tertile 2–3 and activity tertile 2–3	1218.1	30.0	0.71	0.56	0.89
*p* for trend			0.01		
Lifestyle index based on number of risk factors * ^2^					
3–4 risk factors	460.2	26.0	Ref		
2 risk factors	986.4	37.0	0.63	0.36	1.10
1 risk factor	1225.3	45.0	0.74	0.43	1.29
None	160.0	3.0	0.22	0.03	1.71
*p* for trend			0.40		

* Risk factors are estimated for diet, physical activity, smoking, and alcohol use. ^1^ Models were adjusted for age (years), sex (men, women), education level (high school, associate degree, and university), body mass index (kg/m^2^), smoking status (never, former, and current), alcohol use (never, former, and current), activities of daily living (7–35), total energy intake (kilojoule), language background (English, non-English), and comorbidity index. ^2^ Models were adjusted for age (years), sex (men, women), education level (high school, associate degree, and university), body mass index (kg/m^2^), activities of daily living (7–35), total energy intake (kilojoule), language background (English, non-English), snoring (rarely, occasionally, regularly), sleepiness (rarely, slightly dozing, heavy sleepiness), and having a confidant (yes, no), and comorbidity index.

## Data Availability

The data presented in this study are available at the request of the corresponding author and the founders of the Sydney Memory Ageing Study. The data is not publicly available due to privacy or ethical restrictions.

## Supplementary Materials

The following supporting information can be downloaded at: https://www.mdpi.com/article/10.3390/nu17050796/s1, Table S1. Factor loading for each included variable for each lifestyle pattern. Table S2. Lifestyle index based on the Australian health guidelines.
