# Supplementary material for: The Role of Nutrition and Other Lifestyle Patterns in Mortality Risk in Older Adults with Multimorbidity
Source: Nutrients. 2025 Feb 25;17(5):796. doi: 10.3390/nu17050796 (PMC11901584; doi:10.3390/nu17050796)
Supplement: Supplementary file 1 [file nutrients-17-00796-s001.zip › nutrients-3451474-supplementary.pdf]

Supplementary Materials

Supplementary Table S1. Factor loading for the variables retained in each lifestyle pattern

**Variables retained**	**Pattern 1 (Nutrition pattern)**	**Pattern 2 (Exercise-sleep-social pattern)**
Dietary protein intake (g/d)	0.310	0.157
Dietary fibre intake (g/d)	0.300	-0.147
Dietary iron intake (mg/d)	0.320	-0.041
Dietary zinc intake (mg/d)	0.310	0.144
Dietary magnesium intake (mg/d)	0.330	-0.087
Dietary potassium intake (mg/d)	0.320	-0.056
Dietary folate intake (μg/d)	0.300	-0.137
Bowling (minutes/week)	0.020	0.310
Bicycling (minutes/week)	0.057	0.340
Snore (no, moderate, heavy)	0.004	-0.320
Sleepiness (no, slight, heavy)	-0.026	-0.400
Having a confidant (yes, no)	-0.001	0.320
Face-to-face contacts (time/month)	0.015	0.360

Supplementary Table S2. Lifestyle index* based on the Australian health guidelines

**Risk factors**	**Definition**
Poor diet quality	The lowest tertile of meeting the Australian Dietary Guidelines scores
Physical inactivity	Less than 150 minutes of moderate-to-vigorous intensity activity per week
Alcohol use	Over ten standard drinks per week
Tobacco use	Current smoking

*The lifestyle index was defined as 0, 1, 2, 3, or 4 risk factor(s).
